# Supplementary material for: The causal relationship between the human gut microbiota and pyogenic arthritis: a Mendelian randomization study
Source: Front Cell Infect Microbiol. 2024 Nov 26;14:1452480. doi: 10.3389/fcimb.2024.1452480 (PMC11629706; doi:10.3389/fcimb.2024.1452480)
Supplement: Supplementary file 1 [file DataSheet1.zip › mendelian test/ebi-a-GCST90027639.csv_forest.pdf]

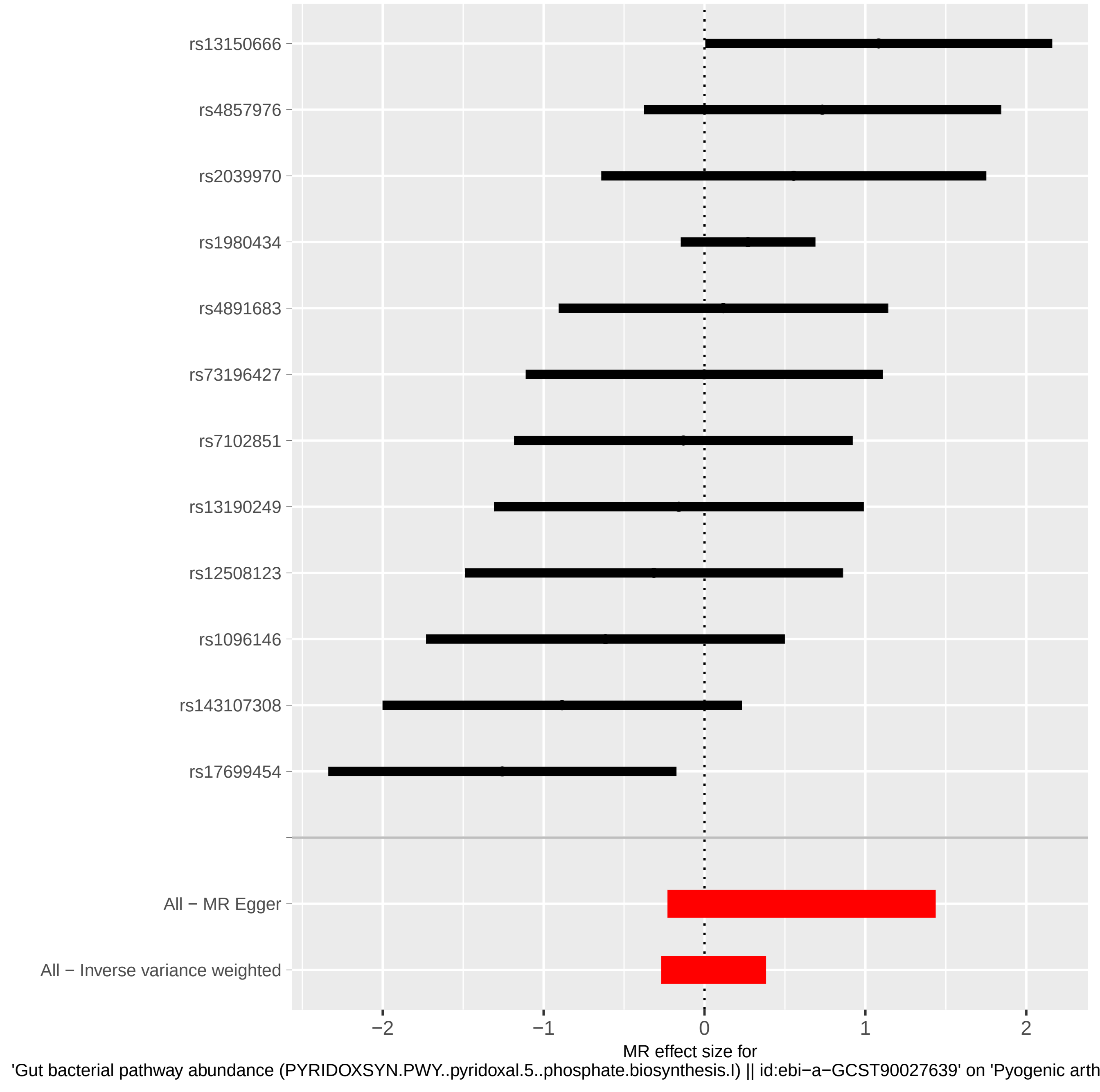
rs13150666
rs4857976
rs2039970
rs1980434
rs4891683
rs73196427
rs7102851
rs13190249
rs12508123
rs1096146
rs143107308
rs17699454
All − MR Egger
All − Inverse variance weighted
−2
−1
0
1
2
MR effect size for
'Gut bacterial pathway abundance (PYRIDOXSYN.PWY..pyridoxal.5..phosphate.biosynthesis.I) || id:ebi−a−GCST90027639' on 'Pyogenic arth
